# Correlation between administered treatment and patient’s living will

**DOI:** 10.3332/ecancer.2009.158

**Published:** 2009-09-29

**Authors:** B Andreoni, A Goldhirsch, R Orecchia, M Venturino, R Spirito, L Tadini, C Corbellini, E Bertani, U Veronesi

**Affiliations:** 1 Division General Surgery, European Institute of Oncology, 20141 Milan, Italy; 2 Department of Medicine, European Institute of Oncology, 20141 Milan, Italy; 3 Division of Radiotherapy, European Institute of Oncology, 20141 Milan, Italy; 4 Division of Anesthesiology, European Institute of Oncology, 20141 Milan, Italy; 5 Scientific Director, European Institute of Oncology, 20141 Milan, Italy; 6 Unit of Vascular Surgery, Centro Cardiologico Monzino, Milano, Italy

## Abstract

Respecting the wishes of an adequately informed patient should be a priority in any health structure. A patient with advanced or terminal cancer should be allowed to express their will during the most important phases of their illness. Unfortunately, this is seldom the case, and in general instructions regarding an individual’s medical care preferences, i.e., their **‘living will’**, expressed when healthy, often change with the onset of a serious illness.

At the European Institute of Oncology (IEO), a clinical study is ongoing to verify whether, during clinical practice, the patient is adequately informed to sign **an ‘informed consent’**, in a fully aware manner, that will allow the patient and doctor to share in the decisions regarding complex treatment strategies (**living will**). A further aim of the study is to verify if health workers, both in hospital and at home, respect the patient’s will.

The observational study ‘Respecting the patient’s wishes: Correlation between administered treatment and that accepted by the patient in their Living Will’ was approved by the IEO Ethical Committee in April 2008.

## Study design

All the patients enrolled in the study will be informed in a way that ensures adequate comprehension (‘giving detailed information’ is not enough; it is necessary to ensure that the patient understands what is involved).

In the case of surgical patients, at the first visit (usually as an outpatient), a research surgeon indicates on the admission form for complex surgery whether the patient fulfils all the inclusion criteria for the study and adequately explains the objectives of the study to the patient for the first time. Subsequently, during the pre-admission visit, the patient re-discusses their complex treatment with the research anaesthesiologist. Finally on the day of admission, patients are asked whether they want to participate in the study previously detailed. If they are interested, a structured interview is organized with the Head of the Division (or other appointed physician), the Head Nurse (or other appointed nurse) and (if the patient consents and the researcher finds it necessary) a family member/friend or general practitioner. If at the end of this interview the patient agrees to participate in the study, they sign an ad-hoc informed-consent form and fill in a questionnaire; research assistants are available to help, should the patient request it. When the patient is discharged, a case report form (CRF) is completed and signed by both the Head of the Division and the Head Nurse.

In the case of medical patients, information regarding advance planning of complex treatments is also given at similar time points, starting with the first visit and finally, if the patient accepts, at the point when he gives his informed opinions in the questionnaire. When the patient is discharged, a case report form is filled in and signed by both the Head of the Division and the Head Nurse/Head Technician.

The patient’s living will is compared to the actual treatments administered as registered on the CRF by the responsible physician at the end of treatment.

## Materials and methods

After being adequately informed, patients sign an ‘informed consent form for the participation in the study’ and fill out a questionnaire at the following ‘critical’ moments:

- after the communication of a diagnosis of serious, advanced, probably incurable cancer;
- before complex surgery;
- before further complex medical treatment.

In order to freely express their consent and to participate in the study, patients must be of sound mind.

## Results

**Figure 1a and b:**
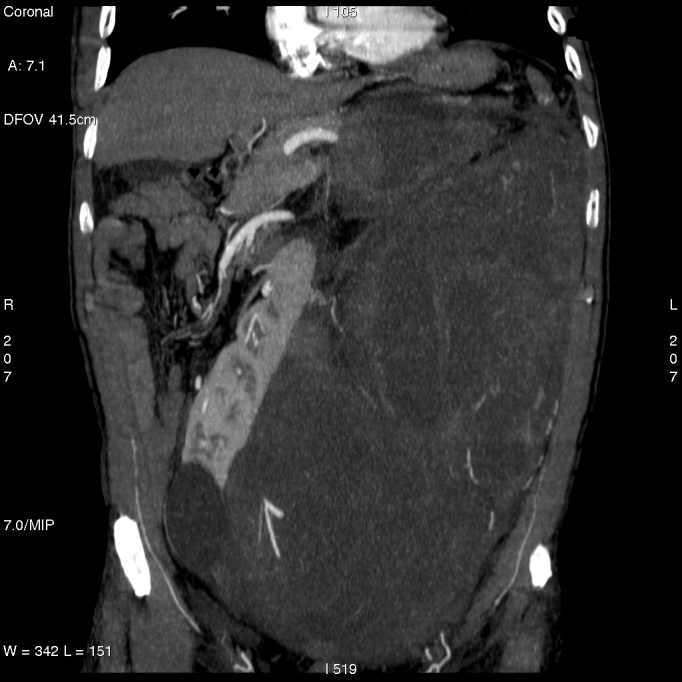

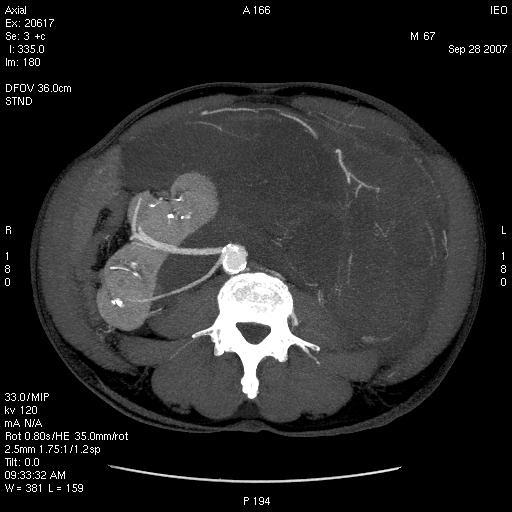
Voluminous retroperitoneal sarcoma in a patient with horseshoe kidney (before surgery)—patient 1.

**Figure 2:**
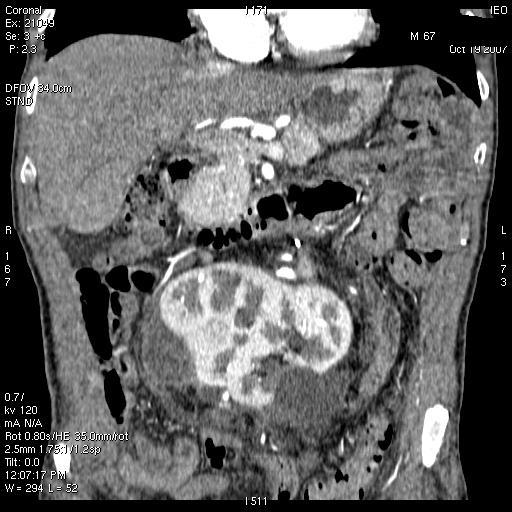
Abdominal CT after surgery—patient 1.

**Figure 3:**
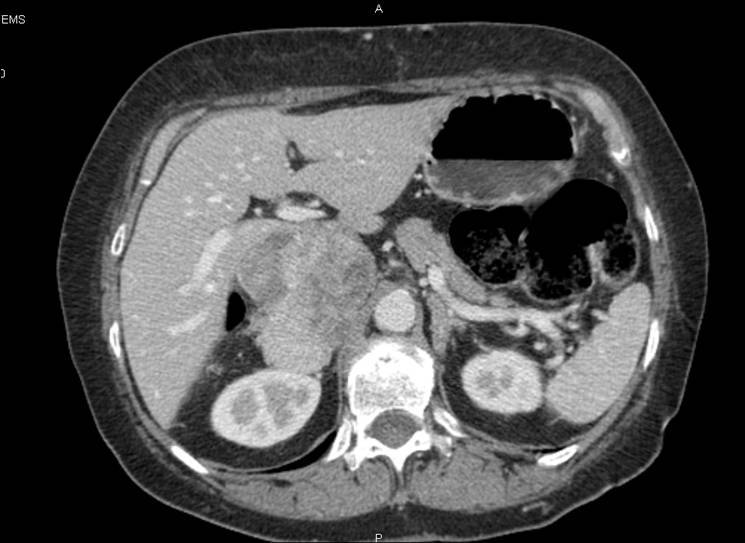
Pre-operative CT—patient 8.

**Figure 4:**
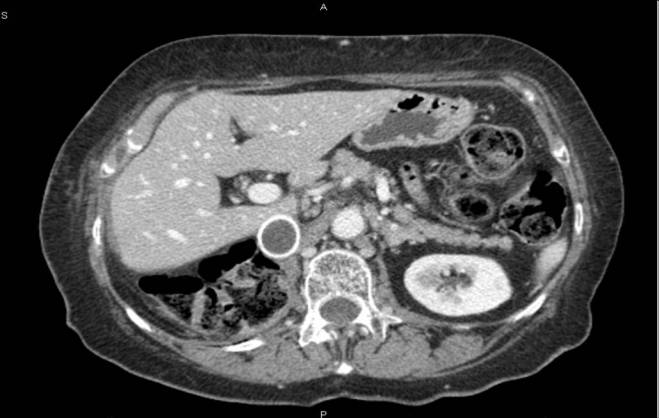
Post-operative CT scan—patient 8

**Figure 5a and b:**
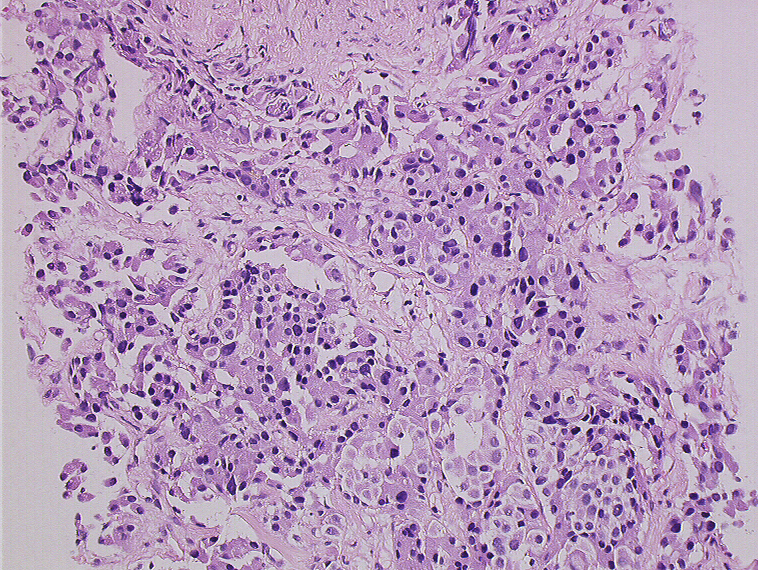

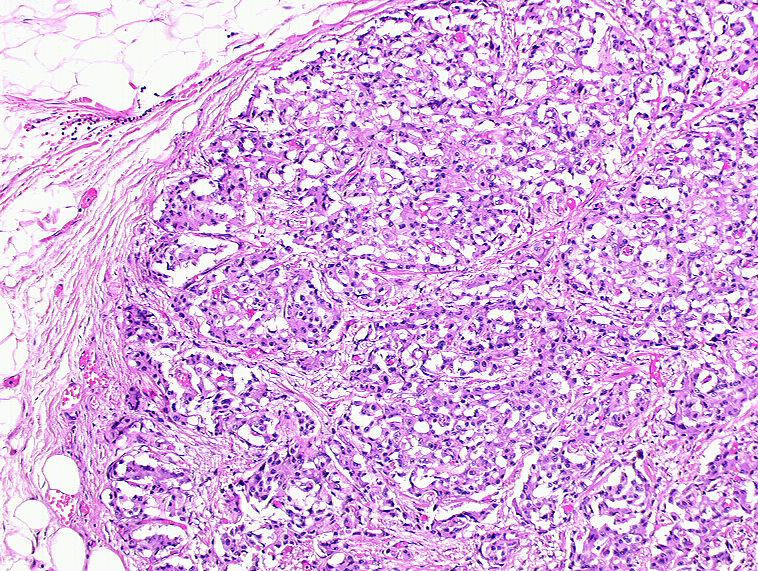
Histology of neuroendocrine tumour—patient 9.

**Figure 6:**
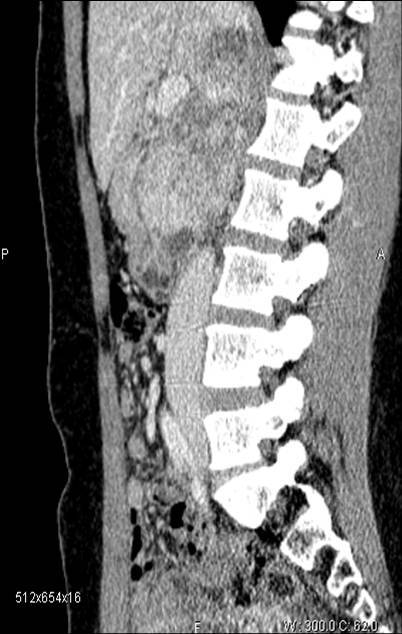
CT scan before surgery—patient 12. Video clip. Surgical procedure of mass resection with cava reconstruction. Patient 12 To view this video click here: http://www.ecancermedicalscience.com/view-article.asp?doi=10.3332/ecancer.2009.158

During the 16 months of the study (February 2008 to June 2009), the following 15 patients were enrolled:

1. A 55-year-old male with voluminous recurrent retroperitoneal sarcoma infiltrating a horseshoe kidney and the colon. Surgery performed: mass resection, partial nephrectomy and colon resection. Discharge after 24 days; at present (after 24 months) the patient is free of disease (see Figure 1a and b and Figure 2).
2. An 86-year-old female with symptomatic liver metastasis to the sixth segment. Surgery: R0 liver resection. Ten-month follow-up: the patient is asymptomatic and free of disease.
3. A 69-year-old female with incisional hernia and peristomal fistula after total pelvectomy for sigmoid cancer infiltrating the bladder. Surgery: hernia repair and re-colostomy.
4. A 55-year-old male with rectal cancer recurrence after previous anterior rectal resection (after neoadjuvant chemoradiotherapy). Surgery: re-resection according to Hartman + Intra-Operative RadioTherapy (IORT).
5. A 55-year-old female diagnosed with distal rectal neoplasia, involving the sphincter with liver and lung metastases. Surgery: robotic Miles abdominal amputation after neoadjuvant chemotherapy.
6. A 70-year-old male diagnosed with pancreatic head cancer (six months before, biliar endoprosthesis for obstructive jaundice). Surgery: cephaloduodenopancreatectomy for pancreatic cancer pT3 pN1.
7. A 35-year-old male Jehovah’s Witness diagnosed with a symptomatic voluminous cystic haemangioma of the second–third liver segment. Surgery: liver resection (without blood supply for transfusion).
8. A 65-year-old female diagnosed with a voluminous retroperitoneal sarcoma behind and below the liver, massively infiltrating the inferior cava vein. Surgery: mass resection with substitution of the inferior cava vein with vascular prosthesis and right nephrectomy (see Figures 3 and 4).
9. A 72-year-old female diagnosed pancreatic head carcinoma without jaundice. Surgery: cephaloduodenopancreatectomy (see Figure 5a and b).
10. A 71-year-old male previously treated with HIFU + external radiotherapy for prostate cancer. Diagnosed with a neoplasia to the distal rectum, the patient underwent Miles abdominal amputation with terminal colostomy.
11. A 59-year-old female diagnosed with a stenosis 4 cm from the anus, with incontinence and tenesmus after colorectal surgery (rectosigmoid resection + liver resection + removal of colonic recurrence). Surgery: Hartmann resection.
12. A 45-year-old female with a symptomatic voluminous retroperitoneal sarcoma massively infiltrating the inferior cava vein, deemed surgically inoperable elsewhere. Surgery: mass resection with cava reconstruction (see Figure 6, video clip).
13. A 56-year-old male diagnosed adenocarcinoma to the caecum and rectum in a patient with familial colonic polyposis and symptomatic brain artero–venous malformation (epileptic crises). Surgery: total proctocolectomy with ileal-anal pouch.
14. A 76-year-old male diagnosed voluminous incisional hernia after total gastrectomy for gastric cancer. Surgery: hernia repair with mesh.
15. A 56-year-old male diagnosed with a local recurrence, after previous emicolectomy, infiltrating the left ureter (with left ureterohydronephrosis) and colon, inseparable from the left iliac vessels. Surgery: recurrence resection with left ureteronephrectomy. The patient died on the seventh day after surgery, just before discharge (probable massive pulmonary embolism).
16. A 63-year-old man diagnosed with stenosing neoplasia to the distal third of the oesophagus, serious malnutrition; aneurism to the abdominal aorta. Surgery: oesophageal resection with oesophago-gastric plastic reconstruction.
17. A 77-year-old male diagnosed with recent haematemesis due to ulcerated gastric cancer; serious myocardiac stroke during hospitalisation for tumour bleeding (strain test with very low threshold because of myocardiac damage). Surgery: total gastrectomy.

## Discussion

While examining the questionnaires filled in by the 15 patients, the researcher group (physicians and nurses from IEO) asked the following questions:

- Were the patients informed correctly?
- Can their wills, as expressed in the questionnaires, be considered ‘informed’ and ‘binding’ for health workers?
- Were their written wills respected at least during treatment at IEO?
- How should physicians and nurses behave in cases of temporary or permanent patient incapacity, when there is disagreement between the treatment intention of the IEO personnel and the patient’s written will?
- How much should the trustee’s opinion, who was chosen by the patient before treatment, count in cases of temporary or permanent patient incapacity?

Pondering these issues certainly helped physicians and nurses involved in the study to grow both from a human and professional point of view.

Both patients and their families appreciated the questions asked in the questionnaire. The meetings with physicians to fill out the questionnaire facilitated sharing choices and established a strong ‘therapeutic alliance’. This allowed constructive communication where patients and their families relied on physicians and nurses who empathically cared for the patient.

In the only adverse case (sudden death of the patient just before hospital discharge), the discussions involved in establishing the living will prior to surgery, helped to create a mutual understanding and support between family and health workers at this difficult time.

In the short lifespan of the IEO, the will of the patient has always been respected. The identification of a trustee, a person close to the patient, has often facilitated the collaboration between the patient and their family and health workers.

The commitment of all clinical researchers involved in the study also led to a revision of the process for the acquisition of informed consent. The previous approach, often limited to a signature on a standard form, has evolved into a more specific procedure aimed at obtaining precise information that will help patients to express their free, informed wishes.
